# Increased levels of NETosis biomarkers in high-grade serous ovarian cancer patients’ biofluids: Potential role in disease diagnosis and management

**DOI:** 10.3389/fimmu.2023.1111344

**Published:** 2023-02-03

**Authors:** Sarai Tomás-Pérez, Julia Oto, Cristina Aghababyan, Raquel Herranz, Aitor Cuadros-Lozano, Eva González-Cantó, Bárbara Mc Cormack, Judith Arrés, María Castaño, Fernando Cana, Laura Martínez-Fernández, Núria Santonja, Rocío Ramírez, Alejandro Herreros-Pomares, Sarai Cañete-Mota, Antoni Llueca, Josep Marí-Alexandre, Pilar Medina, Juan Gilabert-Estellés

**Affiliations:** ^1^ Research Laboratory in Biomarkers in Reproduction, Obstetrics and Gynecology, Research Foundation of the General University Hospital of Valencia, Valencia, Spain; ^2^ Hemostasis, Thrombosis, Arteriosclerosis and Vascular Biology Research Group, Medical Research Institute Hospital La Fe, Valencia, Spain; ^3^ Department of Obstetrics and Gynecology, General University Hospital of Valencia Consortium, Valencia, Spain; ^4^ Department of Statistics and Operational Research, University of Valencia, Valencia, Spain; ^5^ Department of Pathology, General University Hospital of Valencia Consortium, Valencia, Spain; ^6^ Department of Medical Oncology, General University Hospital of Valencia Consortium, Valencia, Spain; ^7^ Department of Biotechnology, Polytechnic University of Valencia, Valencia, Spain; ^8^ Cancer Biomedical Research Network Center, CIBERONC, Madrid, Spain; ^9^ Department of Obstetrics and Gynecology, General University Hospital of Castellon, Castellón, Spain; ^10^ Multidisciplinary Unit of Abdominal Pelvic Oncology Surgery (MUAPOS), General University Hospital of Castellon, Castellón, Spain; ^11^ Department of Medicine, University Jaume I, Castellón, Spain; ^12^ Department of Pediatrics, Obstetrics and Gynecology, University of Valencia, Valencia, Spain

**Keywords:** high-grade serous ovarian cancer, biomarkers, NETosis, cfDNA, calprotectin, peritoneal fluid

## Abstract

**Introduction:**

High-grade serous ovarian cancer (HGSOC) is the second most frequent gynecological malignancy but the most lethal, partially due to the spread of the disease through the peritoneal cavity. Recent evidence has shown that, apart from their role in immune defense through phagocytosis and degranulation, neutrophils are able to participate in cancer progression through the release of neutrophil extracellular traps (NETs) in a process called NETosis. NETs are composed of DNA, histones, calprotectin, myeloperoxidase (MPO) and elastase and the NETosis process has been proposed as a pre-requisite for the establishment of omental metastases in early stages of HGSOC. Nevertheless, its role in advanced stages remains to be elucidated. Therefore, our principal aim is to characterize a NETosis biomarker profile in biofluids from patients with advanced HGSOC and control women.

**Methods:**

Specifically, five biomarkers of NETosis (cell-free DNA (cfDNA), nucleosomes, citrullinated histone 3 (citH3), calprotectin and MPO) were quantified in plasma and peritoneal fluid (PF) samples from patients (n=45) and control women (n=40).

**Results:**

Our results showed that HGSOC patients presented a higher concentration of cfDNA, citH3 and calprotectin in plasma and of all five NETosis biomarkers in PF than control women. Moreover, these biomarkers showed a strong ability to differentiate the two clinical groups. Interestingly, neoadjuvant treatment (NT) seemed to reduce NETosis biomarkers mainly systemically (plasma) compared to the tumor environment (PF).

**Discussion:**

In conclusion, NETosis biomarkers are present in the tumor environment of patients with advanced HGSOC, which might contribute to the progression of the disease. Besides, plasma cfDNA and calprotectin could represent minimally invasive surrogate biomarkers for HGSOC. Finally, NT modifies NETosis biomarkers levels mainly at the systemic level.

## 1 Introduction

Ovarian cancer (OC) is the most lethal gynecologic malignancy (1). The overall survival rate at 5-years follow-up is less than 50% and decreases to 5-21% within 10 years (2). This high mortality can be partly explained by the presence of metastases at the time of diagnosis, its high recurrence rate, and the acquisition of chemoresistance by the tumor (3).

High-grade serous ovarian cancer (HGSOC) is the most frequent and aggressive subtype of OC. It accounts for 70-80% of OC-related deaths, mainly due to its predominant diagnosis in advanced stages, when diffuse widespread peritoneal metastases are already present (1). Although OC metastases may occur *via* systemic or lymphatic routes, most HGSOC tumors spread across the peritoneal cavity following peritoneal fluid (PF) dynamics (4). Malignant accumulation of PF or ascites has been described in >90% HGSOC patients, contributing to chemoresistance, metastasis and poor prognosis (5). Since PF is considered a crucial component of the HGSOC tumor environment (6), an exhaustive characterization of its cellular and molecular components might open new avenues to improve patients’ management.

The gold standard therapy for advanced OC has remained mostly unchanged for decades, consisting of a primary debulking surgery followed by first-line chemotherapy based on platinum and taxanes (7). For patients who are medically unfit to undergo first line surgery or in the presence of unresectable disease, neoadjuvant chemotherapy followed by interval debulking surgery can be considered as an alternative approach (8). Nevertheless, despite advances in the field of surgery and chemotherapy, almost half of patients will develop disease recurrence within 18 months and most of them will die from the disease within 5 years (9). The fact that the 5-year overall survival of advanced OC is 29%, whereas the 5-year OS in early stages is 92% (10) underpins the need for a deeper understanding of the mechanism of disease progression and the evaluation of new therapeutic alternatives. In this context, intraperitoneal chemotherapy, either normo- or hyperthermic, is being considered to improve first line treatment outcomes (11–14).

Novel discoveries in the mechanisms of metastasis and disease progression in cancer have focused on neutrophils and their associated functions (15–17). Neutrophils are the most abundant circulating leukocytes, playing a predominant role in defense mechanisms through phagocytosis and degranulation (18). However, in response to different stimulus (19), neutrophils can release neutrophil extracellular traps (NETs), composed of DNA, histones and cytoplasmic and granular proteins such as calprotectin, myeloperoxidase (MPO) and elastase, in a process called NETosis (20). Since the decondensation of chromatin due to histone 3 citrullination has been described as a key step prior to the formation and release of NETs (21), citrullinated histone 3 (citH3) has been proposed as a specific marker of these structures (22–24). In cancer, NETs may contribute to tumor progression and metastasis (25). Thus, pharmacologically interfering in NET formation or destruction has been recently proposed as a promising therapeutic approach in oncology (26).

Regarding HSOC, recent evidence has proposed NETosis as one of the responsible mechanisms for initial establishment of omental metastases. Lee et al. (27) demonstrated in a murine model that early-stage OC cells can release several factors to recruit neutrophils into the omentum and induce NETosis. Subsequently, disseminated cells through the PF would get trapped into the formed NETs to conform to metastatic implants. However, to the best of our knowledge, the role of NETosis in advanced HGSOC has not been evaluated yet. Hence, our primary aim was to characterize a profile of NETosis biomarkers in biofluids (namely plasma and PF) from patients with advanced HGSOC and control women. Secondarily, we wished to assess its potential diagnostic value and to analyze the possible effect of neoadjuvant treatment (NT) on their levels.

## 2 Materials and methods

### 2.1 Study cohort

The study consists of a retrospective multicenter case-control study. Study subjects were surgically treated at the General University Hospital of Valencia or the General University Hospital of Castellon (Spain). A total of 45 patients diagnosed with advanced HGSOC [IIIC-IV, in accordance with the International Federation of Obstetrics and Gynecology 2014 staging system (28)] and 40 women undergoing tubal sterilization (control group) were recruited.

Exclusion criteria included the presence of infection or neutropenia (neutrophil count<1.5·10^9^/L) in the three weeks prior to surgery and rejection to sign the informed consent. Patients were staged in accordance with the International Federation of Obstetrics and Gynecology 2014 staging system (28). The study was approved by the Institutional Review Board of our institution (protocol code 48/2021, 05/28/2021) and performed according to Ethical Principles of the Declaration of Helsinki and its successive amendments (29).

### 2.2 Clinical-demographic variables

Clinical and demographic data of interest were collected. For both study groups, age, body mass index (BMI), plasmatic neutrophil count and menopausal status were included. For HGSOC patients, performance status (PS) was assessed based on the Eastern Cooperative Oncology Group (ECOG) PS Scale (30) and post-surgical complications were assigned following the Clavien-Dindo classification (31). The mean diameter of the primary tumor was calculated based on computed tomography (CT) image. Peritoneal carcinomatosis index (PCI) was assigned at time of surgery according to the scale established by Jacquet and Sugarbaker (32). In addition, the administration or not of neoadjuvant chemotherapy prior to samples collection was noted.

### 2.3 Sample collection

Blood was withdrawn from all study subjects before induction of anesthesia, collected in Vacuette tubes (Greiner Bio-One) containing 3.2% trisodium citrate and centrifuged at 1800 x g for 30 min at 4°C. Plasma was stored in aliquots at -80°C until used. PF samples were obtained during surgery. Neither prior peritoneal washings were performed nor were anticoagulants used. Fluids with macroscopically visible hemolysis were discarded. Samples were cleared of cells and cell debris by centrifugation at 1500 x g for 30 min at 4°C and stored at -80°C until further use.

### 2.4 Quantification of NETosis biomarkers

Plasma neutrophil count was assessed in DXH900 (Beckman Coulter). Five biomarkers of NETosis were quantified in both biofluids following protocols already employed in our group (33–35). Specifically, cell-free DNA (cfDNA) (Quant-iT PicoGreen dsDNA kit, Life Technologies) and DNA-nucleosomes complexes (Cell Death Detection ELISAPLUS kit, Roche) were measured as markers of neutrophil nuclear content; calprotectin (Human Calprotectin ELISA kit, Hycult Biotech) was measured as marker of cytoplasmic content and MPO (Human MPO ELISA kit, Abnova) as marker of granular content. Finally, DNA- citH3 complexes were also measured, as previously described (36).

### 2.5 Single radial enzyme-diffusion assay

DNaseI activity was assessed in plasma and PF samples with the single radial enzyme-diffusion assay. Agarose gels containing labeled DNA were prepared by dissolving 45 μg/mL salmon sperm DNA (Sigma Aldrich) in a buffer with 20 mM Tris-HCl pH =7.8, 10 mM MgCl2 and 2 mM CaCl2. The solution was heated 10 min at 50°C and mixed with an equal volume of 2% agarose (Condalab) containing 0.08% Safe View (NBS Biologicals) to mark DNA. The mixture was distributed on plates and, after gelification, 1 mm diameter wells were prepared. 2 μL plasma or 4 μL PF were loaded onto the wells in duplicate, and 2 μL of a pool of plasma from 19 healthy controls was included in duplicate in each experiment as inter-assay calibrato. After 17 h incubation at 37°C in a humid chamber, gel degradation halos were observed in a fluorimeter (Uvitec) and photographs were taken. Image J (NIH) was used for the quantification of the degradation halo area.

### 2.6 Statistical analysis

Statistical analyses were performed with the R (version 3.6.2), considering statistically significant a level of p<0.05. All variables were checked for normal distribution using Kolmogorov-Smirnov test. Qualitative variables were summarized as frequency and percentage. Chi-Square test was used to determine association between categorical variables. Quantitative variables were expressed as median and interquartile range. Differences between two independent quantitative variables were assessed using Mann-Whitney U test. The correlation between variables was calculated by Spearman’s rank correlation. Diagnostic cut-off points were analyzed using ROC curves. Linear regression models were performed to assess the possible influence of covariates. Principal component analyses and correlation matrixes were also assessed. Finally, a NETosis score was established considering the individual ability of each PF NETosis biomarker to distinguish HGSOC from control women. Briefly, a cutoff value for each parameter was identified according to the ROC curves Youden’s index. This cutoff was used to dichotomize each parameter in each sample depending on whether the value was above (assigned value: 1) or below (assigned value: 0) the cutoff. Parameters with AUC-ROC curve >0.90 were weighted double as previously described (37). The NETosis score was calculated as follows:


$$\text{NETosis score}=\frac{\sum \text{all parametres}}{\text{maximal score}}\;\times \;100$$

## Results

### 3.1 Study cohort

Plasma and PF samples were analyzed from 45 patients with HGSOC in advanced stages (III-IV) and 40 control women. All women provided plasma samples. Paired PF samples were obtained from 35 (77.8%) of the advanced HGSOC patients and from 21 (52.5%) of the control women. The clinical-demographic characteristics of the study subjects are described in **Table 1**.

**Table 1:** Clinical-demographic characteristics of the study subjects.

	HGSOC patients (n=45)	Control women (n=40)	*p*-value
Age (median; Q1-Q3) (years)	62.0; 51.0-70.3	37.0; 33.3-41.8	<0.001^a^
BMI (median; Q1-Q3) (kg/m^2^)	25.6; 23.9-28.0	25.0; 22.3-30.5	NS^a^
Neutrophil count (median; Q1-Q3) (10^9^/L)	5.3; 3.7-8.4	3.7; 2.8-5.4	0.003^a^
Menopausal status (n (%))			<0.001^b^
Pre-menopause	9 (20.0)	35 (87.5)	
Post-menopause	36 (80.0)	5 (12.5)	
ECOG PS (n (%))			
0	3 (6.7)	NA	
1	24 (53.3)	NA	
2	15 (33.3)	NA	
3	3 (6.7)	NA	
4	0 (0.0)	NA	
5	0 (0.0)	NA	
Post-surgical complications (n (%))			
No complications	31 (68.9)	NA	
I	2 (4.5)	NA	
II	5 (11.1)	NA	
IIIa	0 (0.0)	NA	
IIIb	6 (13.3)	NA	
IVa	0 (0.0)	NA	
IVb	0 (0.0)	NA	
V	1 (2.2)	NA	
Mean primary tumor size (median; Q1-Q3) (mm)	60.5; 44.8-92.1	NA	
PCI (median; Q1-Q3) (AU)	15.0; 7.0-23.3	NA	
Neoadjuvant treatment (n (%))			
Yes	13 (28.9)	NA	
No	32 (71.1)	NA	

BMI, body mass index; ECOG, Eastern Cooperative Oncology Group; HGSOC, high-grade serous ovarian cancer; NA, not applicable; NS, not significant; PCI, peritoneal carcinomatosis index; Q, quartile; SEM, standard error of the mean. ^a^Mann-Whitney U test; ^b^Chi-square test.

Since PF production depends on the hormonal and/or inflammatory influence, a scarcity of PF occurs in post-menopausal women with benign conditions, which hampered the recruitment of a post-menopausal control women cohort. Consequently, we observed significant differences in age and menopausal status between patients and control women. Additionally, plasma neutrophil levels were significantly increased in advanced HGSOC patients. Among patients, 13 (28.3%) received neoadjuvant chemotherapy prior to surgery. The influence of these differences as co-variables on study results is appropriately analyzed hereafter.

Regarding patients, based on the ECOG PS scale, the level of functionality in terms of ability to care for themselves, daily activity and physical ability was >50% (PS≤2) in almost all of them (93.3%). The majority of HGSOC patients (68.9%) did not suffer post-surgical complications. The median mean diameter of the primary tumors was 60.5 mm and median PCI 15.0. Thirteen (28.9%) of the patients had received neoadjuvant chemotherapy prior to surgery.

### 3.2 NETosis biomarkers in PF and plasma of the study subjects

#### 3.2.1 Comparison of NETosis biomarkers between HGSOC patients and control women

**Figure 1:**
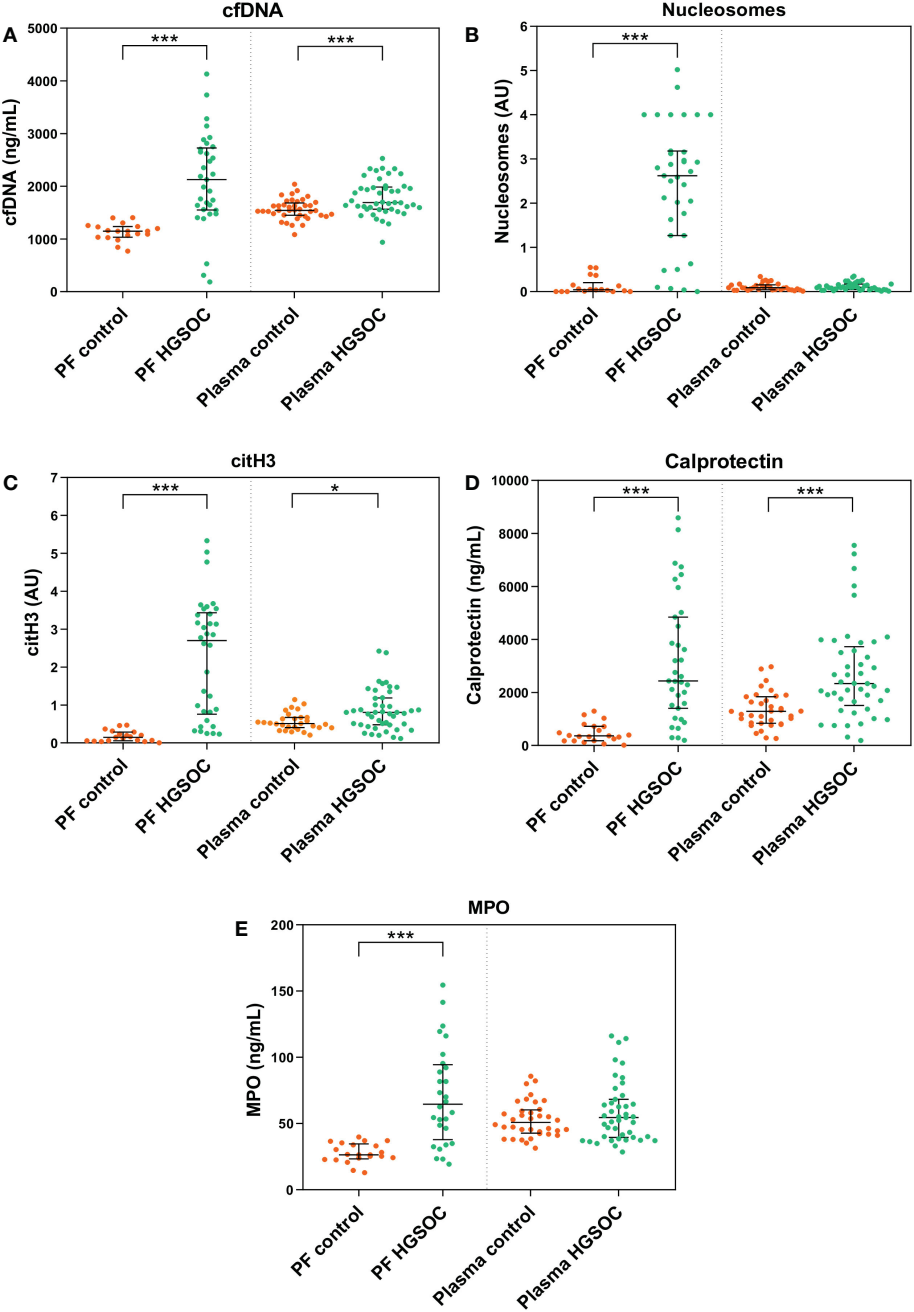
NETosis biomarkers in peritoneal fluid (PF) and plasma samples of patients with high-grade serous ovarian cancer (HGSOC) (n=35 and n=45, respectively) and control women (n=21 and n=40, respectively). **(A)** cell-free DNA (cfDNA). **(B)** Nucleosomes. **(C)** citrullinated histone 3 (citH3). **(D)** Calprotectin. **(E)** Myeloperoxidase (MPO). Median values and interquartile ranges for NETosis biomarkers in both groups are listed in **Supplementary Table S1**. AU, arbitrary units. ***p < 0.001; *p < 0.05; Mann-Whitney U test.

Firstly, we assessed the presence of NETosis biomarkers levels in biofluids of HGSOC patients, suggesting that the contribution of NETosis might not only involve early HGSOC progression. Compared to control women biofluids, HGSOC patients’ PF showed increased levels of all 5 NETosis biomarkers compared to that of control women. In addition, HGSOC patients’ plasma showed elevated levels of cfDNA, citH3 and calprotectin (**Figure 1**).

Nextly, we performed linear regression models to exclude the putative confounding effect that the covariates age, menopausal status and plasma neutrophil count might have produced on the differences in NETosis biomarkers observed between the study groups. We observed that the described differences are not attributable to these covariates, except for menopausal status affecting PF citH3 levels and neutrophil count affecting cfDNA plasma levels (**Supplementary Table S2**).

#### 3.2.2 Suitability of the studied molecules as NETosis biomarkers

To date, no unified marker or detection method has been validated to fully characterize NETosis. Even though H3 citrullination has been described as a characteristic posttranslational modification of NETs; cfDNA, nucleosomes, calprotectin and MPO are also individually expressed, although to a lesser extent, in different cell types. Hence, we assessed the validity of the selected molecules as NETosis biomarkers by means of their correlation in expression, which might point to a common cellular origin.

Considering all study subjects (n=85), a positive correlation was observed among all the 5 NETosis biomarkers in PF (Spearman-ρ≥0.603, p<0.001). In plasma, all molecules correlated pairwise (Spearman- ρ≥0.235, p ≤ 0.037) except for cfDNA with nucleosomes and citH3, and for citH3 and MPO. Remarkably, a significant correlation was observed between plasma neutrophil count and plasma cfDNA and calprotectin (Spearman-ρ≥0.300, p ≤ 0.007) (**Supplementary Table S3**).

Interestingly, sub-analyses per clinical group showed that most of the significant correlations observed among NETosis biomarkers in all study subjects were mainly retained in HGSOC patients but not in control women. Similarly, the correlations between plasma neutrophil count, cfDNA and calprotectin were solely observed in patients (**Supplementary Table S3**).

#### 3.2.3 Performance of the NETosis markers studied as biomarkers of HGSOC

**Figure 2:**
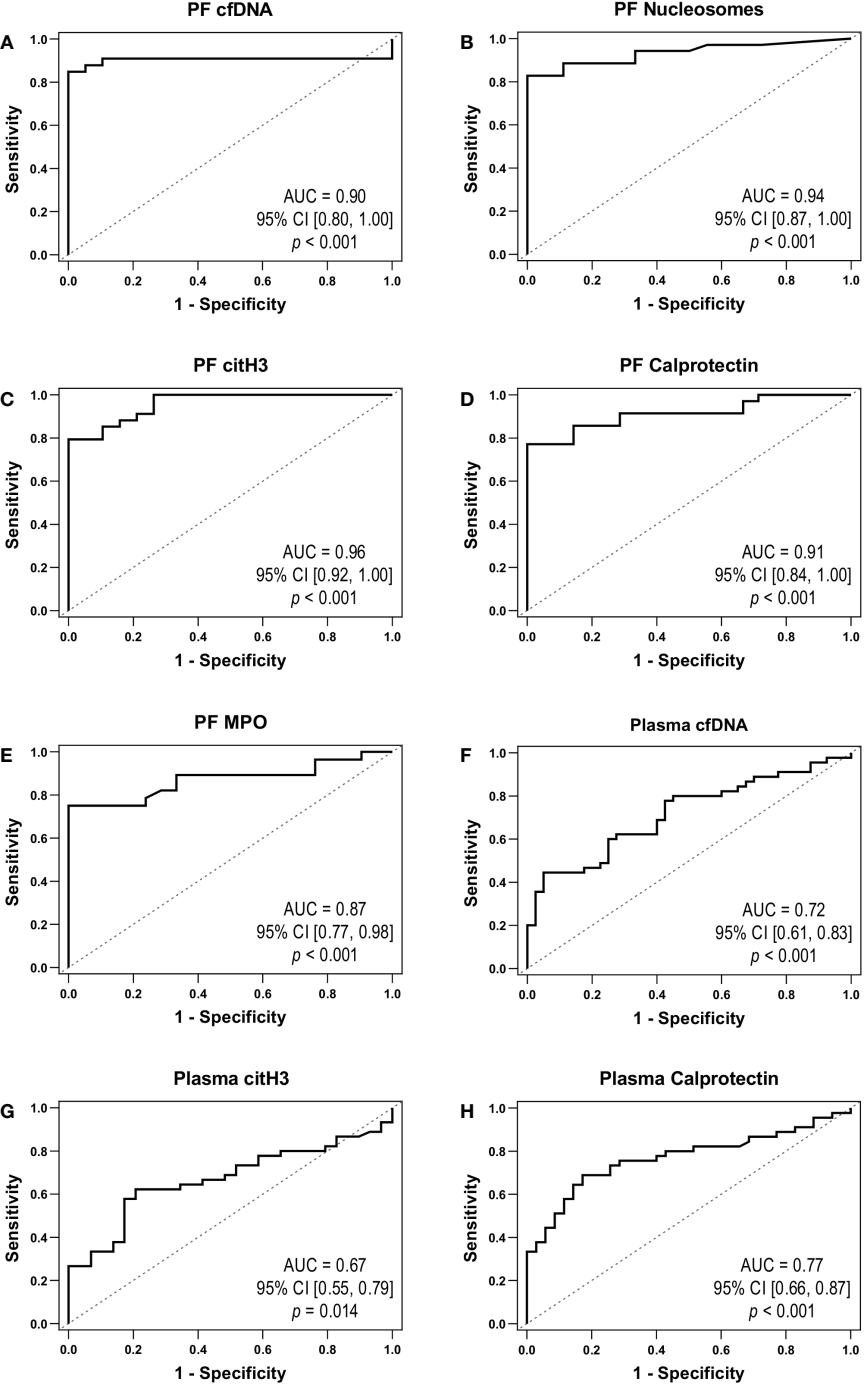
NETosis markers as biomarkers of HGSOC. ROC curves obtained for peritoneal fluid (PF): **(A)** cell-free DNA (cfDNA), **(B)** nucleosomes, **(C)** citrullinated histone 3 (citH3), **(D)** calprotectin and **(E)** myeloperoxidase (MPO). ROC curves obtained for plasma: **(F)** cfDNA, **(G)** citH3 and **(H)** calprotectin. AUC, area under the ROC curve; CI, confidence interval.

We assessed the individual ability of each NETosis marker to distinguish HGSOC patients from control women by performing ROC curves analyses. In line with the aforementioned results, cfDNA, nucleosomes, citH3, calprotectin and MPO in PF (AUC≥0.87; *p*<0.001) (**Figures 2A–E**) and cfDNA, citH3 and calprotectin in plasma (AUC≥0.67, *p ≤* 0.014) (**Figures 2F–H**) clearly differentiated the two clinical groups.

#### 3.2.4 Correlation of NETosis biomarkers between plasma and PF

To gain further insight in the correspondence between the NETosis process in the local tumoral environment (PF) and in the systemic circulation (plasma), we analyzed the correlation of the levels of each biomarker in both biofluids. In HGSOC patients, a positive correlation was for cfDNA (Spearman-ρ=0.765; p<0,001) and calprotectin (Spearman-ρ=0.563; p=0.001) in both biofluids, although not in control women. Remarkably, plasma cfDNA (Spearman-ρ≥0.501, p ≤ 0.001) and calprotectin (Spearman-ρ≥0.429, p ≤ 0.002) positively correlated with the levels of the 5 NETosis biomarkers in PF (**Supplementary Table S4**).

### 3.3 Plasma and PF DNaseI activity

**Figure 3:**
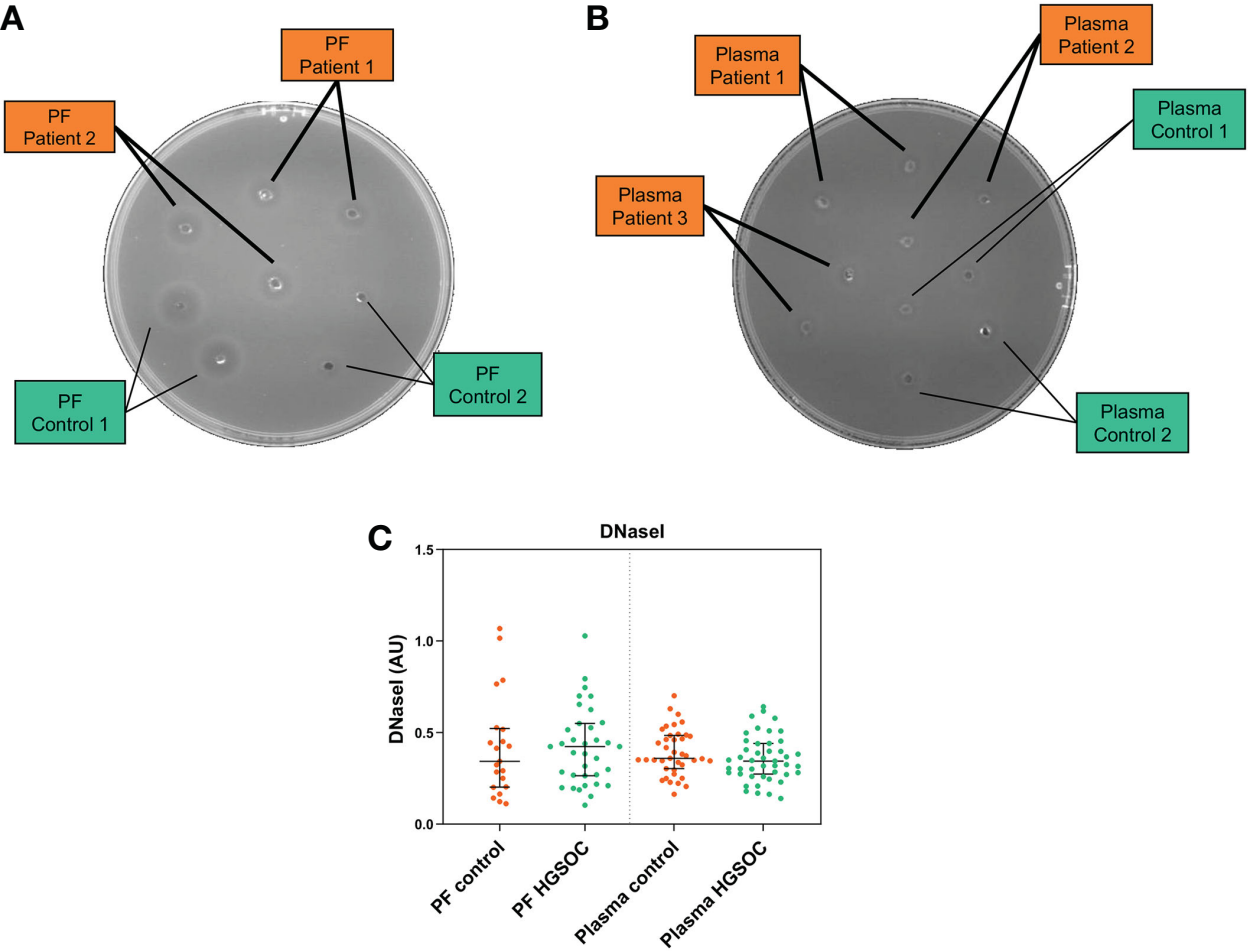
Degradation halo by DNaseI enzyme activity of **(A)** peritoneal fluid (PF) and **(B)** plasma samples of patients with high-grade serous ovarian cancer (HGSOC) (n=35 and n=45, respectively) and control women (n=21 and n=40, respectively) (Single radial enzyme-diffusion assay). **(C)** Qualitative comparison of DNaseI enzyme activity in PF and plasma between clinical groups. AU, arbitrary units. Mann Whitney U test.

One step further, we wished to ascertain whether the increased cfDNA levels in patients’ biofluids (**Figure 1A**) could be attributable to a decreased DNaseI activity between both study groups. To this end, we performed a single radial enzyme-diffusion assay with PF and plasma samples from both study groups. Interestingly, we observed no significant differences in DNaseI activity between clinical groups neither in plasma [(0.36 AU; 0.30-0.49) *vs.* (0.35; 0.27-0.44), NS] nor in PF [(0.34 AU; 0.20-0.52) *vs.* (0.42; 0.26-0.55), NS] (**Figure 3**). The intra-assay coefficient of variation (CV) for PF was 7.5% (n=56) and 0.5% for plasma (n=85), whereas the inter-assay CV obtained with a pool of plasmas in duplicate analyzed in 3 runs over a period of 5 days was 0.9%.

### 3.4 Relationship between NETosis biomarkers and clinical variables

We wished to evaluate the relationship between plasma and PF NETosis biomarkers levels and clinical characteristics of the patients. Specifically, NETosis biomarkers levels were compared and/or correlated to ECOG PS, post-surgical complications, mean primary tumor size and PCI. Analyses showed that there is a lack of relationship between the levels of these markers and the mentioned variables (data not shown).

### 3.5 Influence of neoadjuvant treatment on NETosis biomarkers in HGSOC patients

To ascertain whether the NT might influence the levels of NETosis markers in HGSOC patients, we compared the levels of the 5 biomarkers between HGSOC patients without NT (n=32), HGSOC patients with NT (n=13) and control women (n=40) (**Table 1**).

**Figure 4:**
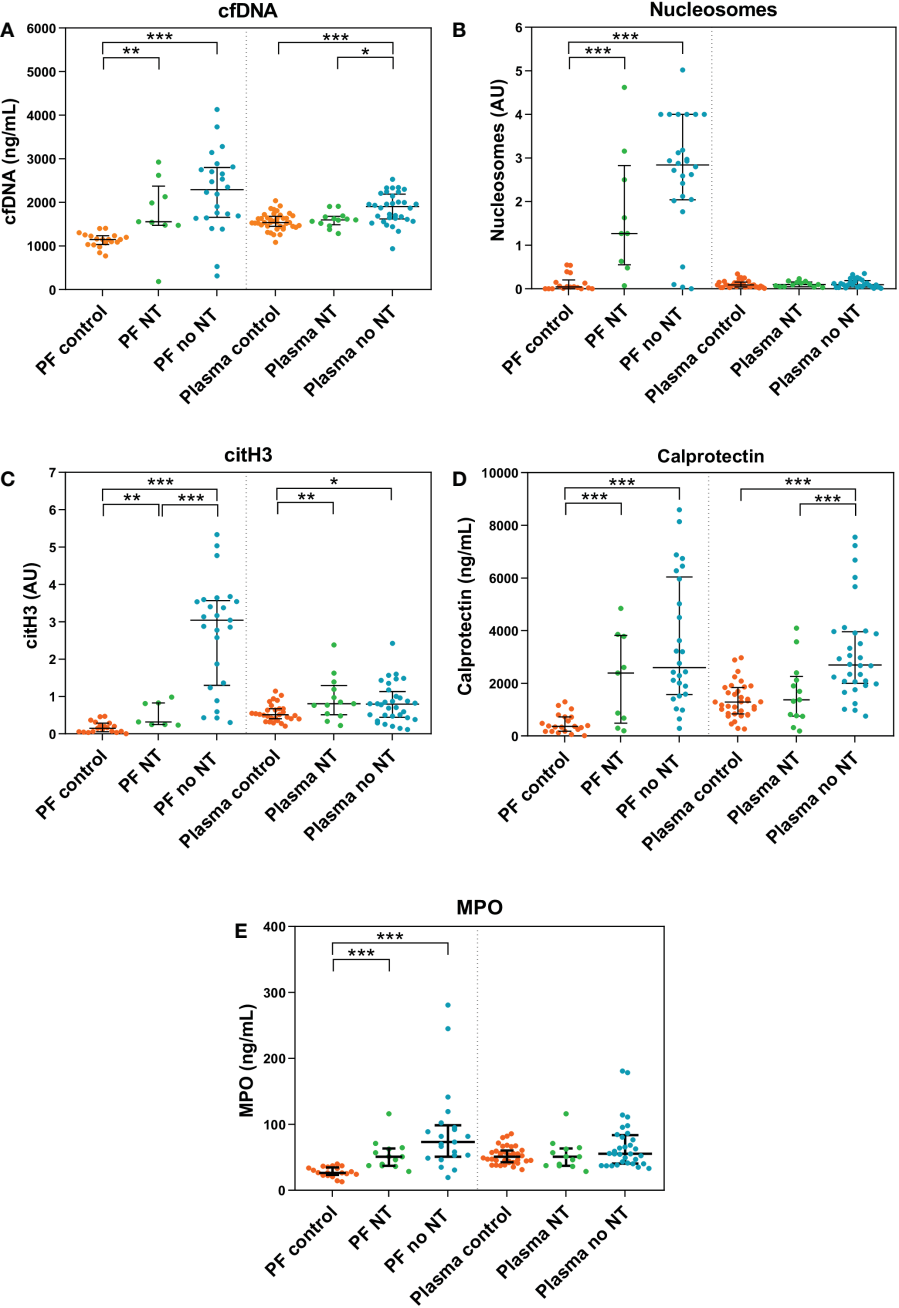
NETosis biomarkers in peritoneal fluid (PF) and plasma samples of patients with high-grade serous ovarian cancer (HGSOC) without neoadjuvant treatment (NT) (n=9 and n=13, respectively), with NT (n=26 and n=32, respectively) and control women (n=21 and n=40, respectively). **(A)** cell-free DNA (cfDNA). **(B)** Nucleosomes. **(C)** citrullinated histone 3 (citH3). **(D)** Calprotectin. **(E)** Myeloperoxidase (MPO). Median values and interquartile ranges for NETosis biomarkers in each group are listed in **Supplementary Table S5**. AU, arbitrary units. ***p < 0.001; **p < 0.01; *p < 0.05; Mann-Whitney U test.

We observed that the levels of all 5 NETosis markers were highly increased in the tumor environment (PF) of HGSOC patients without NT compared to PF of patients with NT. Besides, both groups presented significantly higher levels of NETosis biomarkers in comparison to control women’s PF. Remarkably, PF citH3 was significantly lower in HGSOC with NT than in patients without NT (**Figure 4**).

At the systemic level (plasma), patients without NT also showed increased levels of cfDNA, citH3 and calprotectin than patients with NT and control women. Interestingly, the levels of these 3 significantly decreased in patients with NT, approaching those of control women (**Figure 4**).

#### 3.5.1 Principal component analyses and NETosis score

**Figure 5:**
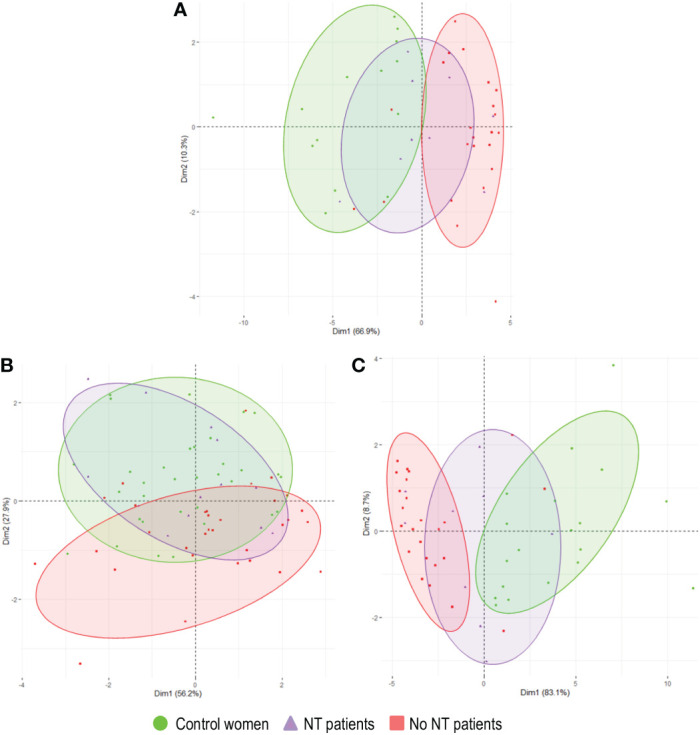
Principal component analyses comparing the levels of the 5 NETosis biomarkers in peritoneal fluid (PF) and plasma samples between control women (n=21 and n=40, respectively), patients with high-grade serous ovarian cancer (HGSOC) who recived neoadjuvant treatment (NT) (n=9 and n=13, respectively) and who did not (n=26 and n=32, respectively). **(A)** NETosis biomarkers in both biofluids, **(B)** in plasma, and **(C)** in PF. Dim, dimension.

Finally, to further characterize the effect of neoadjuvant therapy on NETosis biomarkers, we performed principal component analyses considering the behavior of the 5 NETosis biomarkers in PF and plasma, in the 3 clinical groups (NT patients, no NT patients and control women). In line with our previous results, considering both biofluids (**Figure 5A**) or PF (**Figure 5B**), the graphical algorithm allowed us to differentiate the three clinical groups. Interestingly, in the case of plasma (**Figure 5C**), the analysis closely grouped NT patients and control women.

Additionally, we created a NETosis score to consider the joint behavior of NETosis biomarkers in PF according to their discriminative capacity between clinical groups. Briefly, parameters were dichotomized according to ROC curves Youden’s index. Biomarkers with AUC>0.9 (**Figure 2**) were weighted double. To each patient, NETosis score was calculated as follows:


$$\text{NETosis score}=\frac{\text{cfDNA}\times 0.2+\text{nucleosomes}\times 0.2+citH3\times 0.2+\text{calprotectin}\times 0.2+\text{MPO}\times 0.1}{0.9}\;\times 100$$

**Figure 6:**
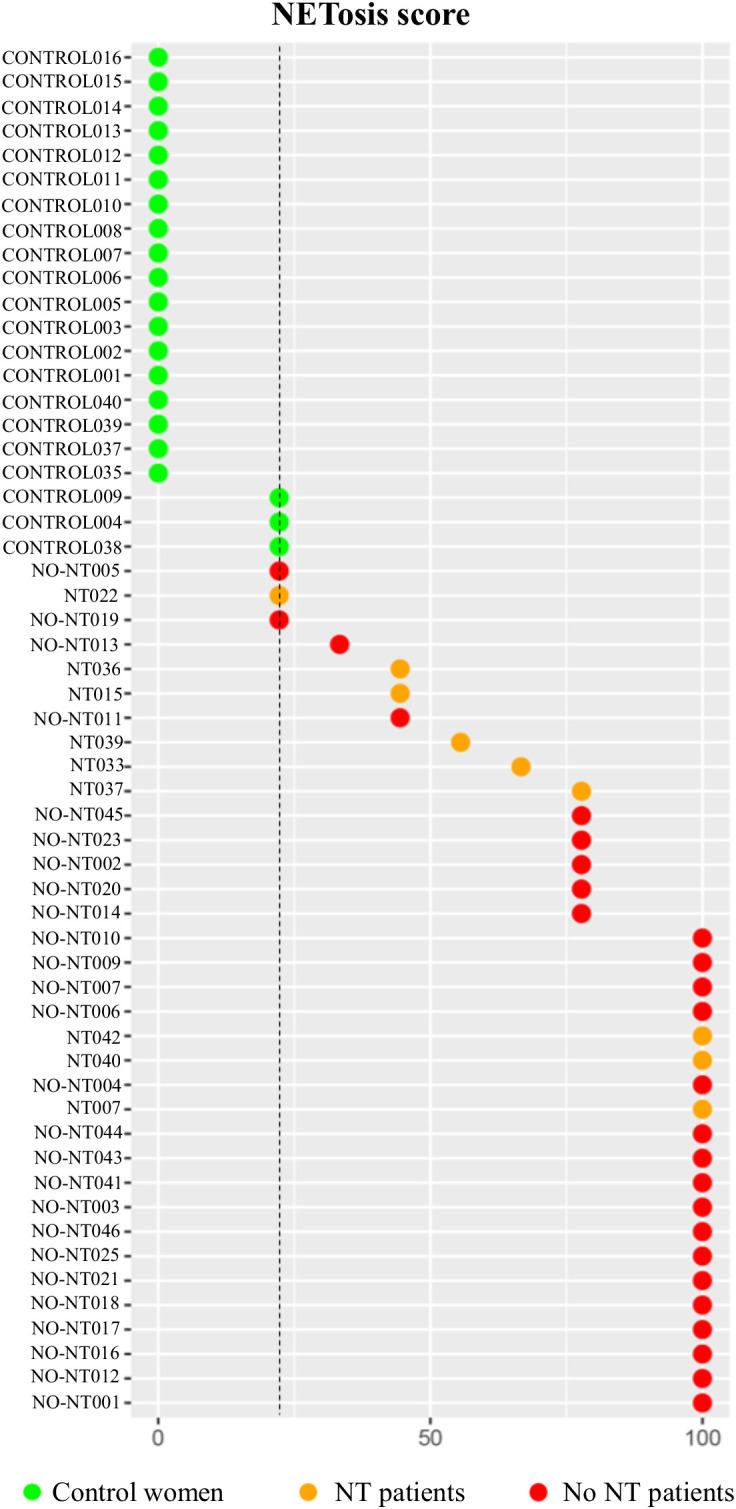
NETosis score values of patients with high-grade serous ovarian cancer (HGSOC) without neoadjuvant treatment (NT) (n=9), with NT (n=26) and control women (n=21). Vertical line represents the NETosis score value (23%) that best allows to differentiate between HGSOC patients and control women. NT, noeadjuvant treatment. *p*<0.001.

A cut-off of 23% was obtained, identifying women with a value<23 as control women, those with a value =23 as undefined and those with a value >23 as HGSOC patients. The NETosis score showed that, concerning the levels of all NETosis biomarkers in PF, HGSOC patients can be clearly differentiated from control women. In agreement with the previous results, it also shows that patients with NT presented decreased levels of NETosis markers in this biofluid, tending to those of control women but without reaching them (**Figure 6**).

## 4 Discussion

HGSOC causes 70-80% of gynecological cancer-related deaths, mainly due to its asymptomatic nature, its diagnosis in advanced stages, the resistance to chemotherapy and the intraperitoneal recurrences (1). Difficulty in the management of affected women (38) highlights the need to better define the biological mechanisms that convert a localized potentially curable disease onto a disseminated fatal disease. In this sense, the contribution of NETosis in cancer is emerging as a hot topic in cancer research (26, 39, 40), although studies in OC in general and in advanced HGSOC, specifically, are scarce. In order to broaden the current knowledge in this field, our principal aim was to characterize a profile of NETosis biomarkers in plasma and PF from patients with advanced HGSOC and control women. Our results show that HGSOC patients have a higher concentration of cfDNA, calprotectin and citH3 in plasma and an increase in the 5 NETosis biomarkers scrutinized (i.e., cfDNA; nucleosomes, citH3, calprotectin and MPO) in PF compared to control women, which would suggest a possible contribution of NETosis in advanced HGSOC. Additionally, our results reveal plasma cfDNA and/or calprotectin as potential minimally invasive surrogate biomarkers for advanced HGSOC. Interestingly, we described that, after neoadjuvant treatment, a significant reduction of NETosis biomarkers occurs mainly systemically but not in the environment where the tumor develops, questioning the efficacy of systemic chemotherapy in the peritoneum, paving the way for alternative therapeutic approaches.

OC predominates in postmenopausal women over 50 years, in which PF accumulation or ascites forms mostly due to local inflammation (41). However, under begin conditions, this biofluid is generated as a mixture of plasma transudate and exudate from ovarian surface tissues under ovarian hormonal influence, predominantly in premenopausal stages (42, 43). Thus, the hormonal and/or inflammatory dependence on PF production has precluded us the recruitment of a control post-menopausal women cohort, resulting in differences in age and menopausal status between our study groups. High counts of blood neutrophils have been documented, as in our case, in OC patients (44), suggesting the key role that this cell type may play in the progression and prognosis of this disease (45–47). Nevertheless, the putative influence of age, menopausal status and neutrophil count as covariates has been excluded in our study using appropriate statistical treatments.

To the best of our knowledge, this is the first study to evaluate the differences in biomarkers of NETosis in biofluids of advanced HGSOC compared to control women. We observed a higher concentration of cfDNA, citH3 and calprotectin in plasma of HGSOC patients and an increase in the levels of the 5 NETosis biomarkers in their PF. Moreover, the subsequent analyses performed to assess the influence of the covariates age, menopausal status and neutrophil count allowed us to confirm that, with the only exception of citH3 in plasma, the differences in the levels of the remaining biomarkers were mainly attributable to the clinical group. As described, the greatest differences mainly occurred in the tumor environment (PF) and not at the systemic level (plasma), which could evidence the contribution of NETosis in the development of HGSOC in the pelvic-abdominal microenvironment.

In a recent work, Lee and coworkers (27) proposed that, under the influence of primary tumor, NETs released by neutrophils on the omentum’s surface serve as a trap for tumor cells migrated into the PF, crucially contributing to the metastasic process in early-stages of HGSOC. Accordingly, we have identified that NETs are present in advanced HGSOC patients’ PF, a biofluid considered a key element of the tumor environment, whose composition crucially conditions the development and progression of the disease (6). Therefore, our findings could suggest that the contribution of NETosis is maintained in advanced stages, converting omental neutrophils and NETs structures into an outstanding therapeutic target. In this regard, the lack of significant direct correlation in our results between NETosis levels and tumor burden (based on tumor size and PCI) might reflect that NETs would be essential for the initial establishment of metastasis at sites free of tumors. However, once the tumor cells have established a metastatic niche (potentially with an optimal blood supply, extracellular matrix invasion, etc.) the function of NETs would not be necessary to maintain the metastatic niche progression. Future studies in patients’ serial samples may shed light on the kinetics of NETs formation and its role in HGSOC progression. Although current literature describes NETs formation as a brief process that occurs in a short time interval (35, 48), analyses in patients’ serial samples might clarify whether NETosis activation in advanced HGSOC patients is a gradually increasing process or, on the contrary, presents an “all/nothing activation”. Besides, it has to be taken into account that PCI assessment depends on the visual acuity of the surgeon, as it evaluates the macroscopic disease, which could underestimate those initial metastatic niches areas with microscopic disease. Furthermore, the quantification of NETosis markers in tumor and omental samples from advanced HGSOC patients might favor a broader understanding of the contribution of NETosis in advanced HGSOC progression.

A challenge in NETosis research is represented by the selection of the best biomarker, since no standard marker or direct and simple method has been validated for the detection of NETs (49) and differences exist in terms of specificity, objectivity or quantification. Although in our work we have quantified the levels of citH3, which has been proposed as a specific marker of this process structures (22–24), the remaining molecules measured herein as markers of NETosis (cfDNA, nucleosomes, calprotectin and MPO) could have a cellular origin other than neutrophils. Specifically, calprotectin and MPO are also expressed, to a lesser extent, in monocytes, macrophages or eosinophils (50, 51); and cfDNA and nucleosomes can be released by apoptotic or necrotic cells present in cancer patients (52). In our hands, the levels of all 5 analyzed biomolecules correlated pairwise, mainly in PF’ HGSOC patients, reinforcing a potential common origin and their suitability as NETosis biomarkers. The different behavior noted for the molecules studied in both biofluids could suggest that the increased levels observed in HGSOC’ PF compared to control women might be attributed to a greater activation of NETosis process in the tumor environment. On the contrary, the differences observed in plasma could be attributable either to NETosis or other processes in which these molecules are involved.

Subsequently, we assessed the potential of the molecules studied as biomarkers of advanced HGSOC. ROC curve analyses showed a high accuracy for the 5 NETosis biomarkers in PF (AUC×0.87) and cfDNA, citH3 and calprotectin in plasma (AUC×0.67). Interestingly, the positive correlation of plasma cfDNA and calprotectin and the levels of the 5 NETosis biomarkers in PF, suggest the quantification of these makers in plasma as a minimal invasive determination potentially informative of the increased NETosis in the tumor environment.

A number of studies have proposed plasma cfDNA as a novel marker of OC (53). On the other hand, most of the studies focused on the potential role of calprotectin as biomarker are based on its fecal determination (54), without promising results in cancer in general (55, 56) and in OC in particular; and with limited studies in plasma calprotectin levels in cancer (57). Remarkably, Odegaard et al. (58) described an increase in circulating calprotectin in patients with OC, suggesting, in line with us, its possible use as a clinical tool, although further studies are required.

In line with our results, decreased DNaseI activity has been associated with increased levels of NETs in some disorders such as lupus erythematosus (59) and acute thrombotic microangiopathy (60). However, this is not responsible for the increase in NETosis markers in our HGSOC patients as similar DNaseI activity levels were detected than controls both in plasma and PF.

Regarding HGSOC therapeutic management, our results showed that biomarkers of NETosis decrease as a consequence of neoadjuvant chemotherapy, tending to those of the control group, predominantly in plasma but not in PF. This observation might imply that, at least for the NETosis process, the intravenous administration of neoadjuvant chemotherapy would produce more changes at the systemic level than in the peritoneal tumor environment, which could support the potential usefulness of intraperitoneal chemotherapy in OC. Besides, it has been reported that neutrophils reach the omentum *via* specialized high endothelial venules in inflammatory conditions of the peritoneum, from where they migrate to the surface area and extrude NETs that remain exposed into the peritoneal cavity (61). As far as OC is concerned, this location of NETs would mean an absence of close contact between tumor cells and the systemic circulation in early stages of metastasis. Therefore, systemic administration of chemotherapy may reach adequate cytotoxic effects in the well vascularized primary tumors, but might be insufficient for the recently migrated NETs-trapped ovarian cancer cells on the omental surface with an incipient vascularization *via* angiogenesis. Altogether, the clinical derivative of our findings could partially explain the intraperitoneal recurrences of HGSOC despite proper surgical and chemotherapy treatment and could envisage a benefit for the use of intraperitoneal chemotherapy for the microscopic disease (62, 63), which may be improved with the incorporation of NETs inhibitors or degradation components.

The difficulty in obtaining PF samples from healthy postmenopausal women is one of the main limitations of our study. However, devoted statistical analyses have been performed to rule out the possible effect of these covariates. The sample size studied is rather limited. Nonetheless, the selection of patients following the established exclusion criteria and the difficulty in recruiting paired samples of the biofluids of interest (maintly PF) hinders the recruitment and management of a high number of patients for this type of studies. In addition, the potential of plasma cfDNA and calprotectin as biomarkers of HGSOC ought to be tested in larger independent cohorts and for early-stages HGSOC patients, for which effective diagnostic approaches have not been established yet. Finally, other studies designed to characterize the NETosis process and/or identify its role in HGSOC would be necessary.

Our results provide a proof of concept of the activation of the NETosis process in the tumor environment of patients with advanced HGSOC. Nevertheless, the research of the contribution of this process in OC is still at its infancy and several factors are yet to be deeply studied, as the association between plasma and omental neutrophil count, half-life time of omental neutrophils and the stoichiometry between neutrophils, NETs and tumor cells to establish the lower number of neutrophils capable of promoting metastasis as well as the exhaustive characterization of such interaction, to mention a few.

## 5 Conclusion

In conclusion, the present study represents a proof of concept on the alteration of the NETosis biomarkers in patients with advanced HGSOC and their potential implications in patients’ management. The positive correlations obtained in both biofluids and clinical groups indicate that the analyzed biomarkers could be useful as NETosis biomarkers in advanced HGSOC. Our results suggest that an increased NETosis occurs in biofluids from HGSOC patients, mainly at the tumor environment (PF) in comparison to the systemic level (plasma), potentially contributing to the progression of HGSOC in advanced stages. The correlation between PF and plasma levels of cfDNA and calprotectin might postulate these molecules as potential low-invasive biomarkers of HGSOC that may improve current diagnostic markers. Finally, we observed that the systemic neoadjuvant treatment has a major influence on NETosis at the systemic levels but its effect is rather limited in the tumor environment, which might improve the therapeutic landscape of HGSOC.

## Data availability statement

The raw data supporting the conclusions of this article will be made available by the authors, without undue reservation.

## Ethics statement

The studies involving human participants were reviewed and approved by Ethics Committee, Research Foundation of the General University Hospital of Valencia, protocol code 48/2021, 28/05/2021. The patients/participants provided their written informed consent to participate in this study.

## Author contributions

JM-A, PM and JG-E contributed to the conception and design of the study. CA, AC-L, FC, LM-F, NS, RR, SC-M and AL achieved and organized a clinical data base. ST-P, JO, RH and EG-C performed the experiments. ST-P, JA, MC, and AH-P performed the statistical analysis. ST-P, JO, EG-C, JA and PM visualized and interpreted the results. ST-P and JM-A wrote the first draft of the manuscript. JO, BM, MC, AL, PM and JG-E reviewed and edited the manuscript. JM-A, PM and JG-E supervised and acquired resources and funding. All authors contributed to the article and approved the submitted version.
