# Supplementary material for: Increased levels of NETosis biomarkers in high-grade serous ovarian cancer patients’ biofluids: Potential role in disease diagnosis and management
Source: Front Immunol. 2023 Feb 3;14:1111344. doi: 10.3389/fimmu.2023.1111344 (PMC9936152; doi:10.3389/fimmu.2023.1111344)
Supplement: Supplementary file 1 [file Table_1.docx]

**Supplementary Table 1. Comparison of NETosis biomarkers in peritoneal fluid (PF) and plasma samples of patients with high-grade serous ovarian cancer (HGSOC) (n=35 and n=40, respectively) and control women (n=21 and n=40, respectively).** Values are expressed as median and interquantile (Q) range (median; Q1-Q3). AU, arbitrary units; cfDNA, cell-free DNA; citH3, citrullinated histone 3; MPO, myeloperoxidase; NS, not significative; NT, neoadjuvant treatment; PF, peritoneal fluid. Mann-Whitney U test.

|  | **PF** | | |
| --- | --- | --- | --- |
|  | **Control women** | **HGSOC patients** | ***p-*value** |
| **cfDNA (ng/mL)** | 1148.2; 1035.2-1237.8 | 2128.9; 1551.2-2728.3 | **<0.001** |
| **Nucleosomes (AU)** | 0.05; 0.00-0.21 | 2.62; 1.27-3.18 | **<0.001** |
| **citH3 (AU)** | 0.020; 0.06-0.46 | 2.70; 0.76-3.43 | **<0.001** |
| **Calprotectin (ng/mL)** | 364.3; 176.8-719.8 | 2434.2, 1406.6-4844.4 | **<0.001** |
| **MPO (ng/mL)** | 26.4; 23.7-34.6 | 64.6; 37.8-94.5 | **<0.001** |
|  | **Plasma** | | |
|  | **Control women** | **HGSOC patients** | ***p*-value** |
| **cfDNA (ng/mL)** | 1541.1; 1452.0-1683.3 | 1690.5; 1565.7-1986.5 | **<0.001** |
| **Nucleosomes (AU)** | 0.09; 0.05-0.16 | 0.10; 0.05-0.17 | NS |
| **citH3 (AU)** | 0.51; 0.40-0.67 | 0.81; 0.48-1.18 | **0.014** |
| **Calprotectin (ng/mL)** | 1286.1; 836.0-1835.8 | 2336.4; 1512.3-3730.0 | **<0.001** |
| **MPO (ng/mL)** | 50.8; 42.7-60.4 | 54.4; 39.5-68.3 | NS |
