# Supplementary material for: Increased levels of NETosis biomarkers in high-grade serous ovarian cancer patients’ biofluids: Potential role in disease diagnosis and management
Source: Front Immunol. 2023 Feb 3;14:1111344. doi: 10.3389/fimmu.2023.1111344 (PMC9936152; doi:10.3389/fimmu.2023.1111344)
Supplement: Supplementary file 2 [file Table_2.docx]

**Supplementary Table 2. Analysis of covariates for the differences in NETosis biomarkers of interest in (A) PF and (B) plasma.** Data is expressed as *p*-values. cfDNA, cell-free DNA; citH3, citrullinated histone 3; MPO, myeloperoxidase; PF: peritoneal fluid. Linear regression models.

| **A** |  |  | |  |  |  |  |  |
| --- | --- | --- | --- | --- | --- | --- | --- | --- |
|  |  | | | **Peritoneal Fluid** | | | | |
|  |  |  |  | **cfDNA** | **Nucleosomes** | **citH3** | **Calprotectin** | **MPO** |
|  |  |  |  | ***p-*value** | | | | |
|  | Fixed factor | | Clincal group | **0.016** | **0.007** | **<0.001** | **0.041** | **0.004** |
|  | Covariates | | Age | 0.633 | 0.757 | 0.185 | 0.623 | 0.587 |
|  |  |  | Menopausal status | 0.675 | 0.496 | **0.031** | 0.859 | 0.586 |
|  |  |  | Neutrophil count | 0.590 | 0.471 | 0.601 | 0.491 | 0.625 |
|  | Linear Regression Model | | | <0.001 | <0.001 | <0.001 | <0.001 | <0.001 |
|  |  | | | **R Squared** | | | | |
|  | Linear Regression Model | | | 0.855 | 0.748 | 0.712 | 0.636 | 0.797 |
|  |  |  | |  |  |  |  |  |
| **B** |  |  | |  |  |  |  |  |
|  |  | | | **Plasma** | | | | |
|  |  |  |  | **cfDNA** | **Nucleosomes** | **citH3** | **Calprotectin** | **MPO** |
|  |  |  |  | ***p-*value** | | | | |
|  | Fixed factor | Clincal group | | **<0.001** |  | 0.461 | **0.013** |  |
|  | Covariates | Age | | 0.667 |  | 0.812 | 0.999 |  |
|  |  | Menopausal status | | 0.856 |  | 0.327 | 0.870 |  |
|  |  | Neutrophil count | | **0.046** |  | 0.088 | 0.072 |  |
|  | Linear Regression Model | | | <0.001 |  | <0.001 | <0.001 |  |
|  |  | | | **R Squared** | | | | |
|  | Linear Regression Model | | | 0.972 |  | 0.753 | 0.725 |  |
