# Supplementary material for: Increased levels of NETosis biomarkers in high-grade serous ovarian cancer patients’ biofluids: Potential role in disease diagnosis and management
Source: Front Immunol. 2023 Feb 3;14:1111344. doi: 10.3389/fimmu.2023.1111344 (PMC9936152; doi:10.3389/fimmu.2023.1111344)
Supplement: Supplementary file 3 [file Table_3.docx]

|  | **All study subjects** | | | | | | | | | | |
| --- | --- | --- | --- | --- | --- | --- | --- | --- | --- | --- | --- |
|  | **PF (n=56)** | | | | | **Plasma (n=85)** | | | | | |
|  | cfDNA | Nucleosomes | citH3 | Calprotectin | MPO | cfDNA | Nucleosomes | citH3 | Calprotectin | MPO | Neutrophil count |
| cfDNA | 1.000 | **0.720***** | **0.603***** | **0.763***** | **0.753***** | 1.000 | 0.098 | 0.111 | **0.596***** | **0.289**** | **0.300**** |
| Nucleosomes |  | 1.000 | **0.692***** | **0.656***** | **0.647***** |  | 1.000 | **0.540***** | **0.276*** | **0.294*** | 0.234 |
| citH3 |  |  | 1.000 | **0.682***** | **0.609***** |  |  | 1.000 | **0.328**** | 0,174 | 0.228 |
| Calprotectin |  |  |  | 1.000 | **0.875***** |  |  |  | 1.000 | **0.476***** | **0.421**** |
| MPO |  |  |  |  | 1.000 |  |  |  |  | 1.000 | 0.195 |
| Neutrophil count |  |  |  |  |  |  |  |  |  |  | 1.000 |
|  | **HGSOC patients** | | | | | | | | | | |
|  | **PF (n=35)** | | | | | **Plasma (n=45)** | | | | | |
|  | cfDNA | Nucleosomes | citH3 | Calprotectin | MPO | cfDNA | Nucleosomes | citH3 | Calprotectin | MPO | Neutrophil count |
| cfDNA | 1.000 | **0.597***** | **0.516**** | **0.571**** | **0.534**** | 1.000 | 0.208 | 0.013 | **0.606***** | 0.290 | **0.301*** |
| Nucleosomes |  | 1.000 | **0.350*** | 0.299 | **0.455*** |  | 1.000 | **0.717***** | **0.357*** | **0.377*** | 0.284 |
| citH3 |  |  | 1.000 | **0.413*** | 0.258 |  |  | 1.000 | **0.306*** | 0.171 | 0.236 |
| Calprotectin |  |  |  | 1.000 | **0.871***** |  |  |  | 1.000 | **0.491**** | **0.381*** |
| MPO |  |  |  |  | 1.000 |  |  |  |  | 1.000 | 0.279 |
| Neutrophil count |  |  |  |  |  |  |  |  |  |  | 1.000 |
|  | **Control women** | | | | | | | | | | |
|  | **PF (n=21)** | | | | | **Plasma (n=40)** | | | | | |
|  | cfDNA | Nucleosomes | citH3 | Calprotectin | MPO | cfDNA | Nucleosomes | citH3 | Calprotectin | MPO | Neutrophil count |
| cfDNA | 1.000 | -0.242 | -0.435 | 0.311 | 0.133 | 1.000 | -0.080 | -0.029 | **0.382*** | **0.382*** | 0.147 |
| Nucleosomes |  | 1.000 | **0.687**** | 0.281 | 0.169 |  | 1.000 | 0.171 | 0.116 | 0.122 | 0.166 |
| citH3 |  |  | 1.000 | 0.225 | 0.389 |  |  | 1.000 | 0.045 | 0.083 | 0.058 |
| Calprotectin |  |  |  | 1.000 | **0.484*** |  |  |  | 1.000 | **0.451**** | 0.166 |
| MPO |  |  |  |  | 1.000 |  |  |  |  | 1.000 | 0.21 |
| Neutrophil count |  |  |  |  |  |  |  |  |  |  | 1.000 |

**Supplementary Table S3. Spearman’s rho correlation coefficients and *p-*values for the correlations of NETosis biomarkers in PF and plasma samples of all study subjects (n=56 and n=85, respectively), HGSOC patients (n=45 and n=35, respectively) and control women (n=21 and n=40, respectively).** cfDNA, cell-free DNA; citH3, citrullinated histone 3; MPO, myeloperoxidase; n, sample size; PF, peritoneal fluid. *** p<0.001; ** p<0.01; * p<0.05. Spearman’s rank correlation.
