# Supplementary material for: Increased levels of NETosis biomarkers in high-grade serous ovarian cancer patients’ biofluids: Potential role in disease diagnosis and management
Source: Front Immunol. 2023 Feb 3;14:1111344. doi: 10.3389/fimmu.2023.1111344 (PMC9936152; doi:10.3389/fimmu.2023.1111344)
Supplement: Supplementary file 4 [file Table_4.docx]

|  |  | **PF** | | | | |
| --- | --- | --- | --- | --- | --- | --- |
|  |  | **cfDNA** | **Nucleosomes** | **citH3** | **Calprotectin** | **MPO** |
| **Plasma** | **cfDNA** | **0.765***** | **0.346*** | **0.476**** | **0.444*** | **0.496**** |
|  | **Calprotectin** | **0.518**** | 0.220 | **0.653**** | **0.413*** | **0.517**** |

**Supplementary Table S4. Spearman’s rho correlation coefficients and *p-*values for the correlations between the 5 NETosis biomarkers’ levels in PF and plasma cfDNA and calprotectin in HGSOC patients (n=35).** cfDNA, cell-free DNA; citH3, citrullinated histone 3; MPO, myeloperoxidase; PF: peritoneal fluid. *** p<0.001; ** p<0.01; * p<0.05. Spearman’s rank correlation.
