# Supplementary material for: Increased levels of NETosis biomarkers in high-grade serous ovarian cancer patients’ biofluids: Potential role in disease diagnosis and management
Source: Front Immunol. 2023 Feb 3;14:1111344. doi: 10.3389/fimmu.2023.1111344 (PMC9936152; doi:10.3389/fimmu.2023.1111344)
Supplement: Supplementary file 5 [file Table_5.docx]

**Supplementary Table S5. Comparison of NETosis biomarkers in peritoneal fluid (PF) and plasma samples of patients with high-grade serous ovarian cancer (HGSOC) with neoadjuvant treatment (NT) (n=9 and n=13, respectively), without NT (n=26 and n=32, respectively) and control women (n=21 and n=40, respectively).** Values are expressed as median and interquantile (Q) range (median; Q1-Q3). cfDNA, cell-free DNA; citH3, citrullinated histone 3; MPO, myeloperoxidase; NS, not significative; NT, neoadjuvant treatment; PF, peritoneal fluid. Mann-Whitney U test.

|  | **PF** | | | | | |
| --- | --- | --- | --- | --- | --- | --- |
|  | **A** | **B** | **C** | **A *vs. B*** | **A *vs.* C** | **B *vs.* C** |
|  | **Control women (n=21)** | **NT patients (n=9)** | **No NT patients (n=26)** |  |  |  |
| **cfDNA (ng/mL)** | 1148.2; 1035.2-1237.8 | 1556.5; 1475.6-2375.4 | 2292.3; 1656.1-2798.5 | **0.002** | **<0.001** | NS |
| **Nucleosomes (AU)** | 0.05; 0.00-0.21 | 1.27; 0.56-2.83 | 2.84; 2.04-4.00 | **<0.001** | **<0.001** | NS |
| **citH3 (AU)** | 0.15; 0.05-0.28 | 0.32; 0.25-0.83 | 3.04; 1.30; 3.57 | **0.004** | **<0.001** | **<0.001** |
| **Calprotectin (ng/mL)** | 364.3; 176.6-719.8 | 2390.8; 484.7-3817.1 | 2594.7; 1568.1-6037.8 | **<0.001** | **<0.001** | NS |
| **MPO (ng/mL)** | 26.4; 23.4-34.6 | 50.9; 37.2-63.6 | 73.3; 50.1-98.8 | **<0.001** | **<0.001** | NS |
|  | **Plasma** | | | | | |
|  | **A** | **B** | **C** | **A *vs. B*** | **A *vs.* C** | **B *vs*. C** |
|  | **Control women (n=40)** | **NT patients (n=13)** | **No NT patients (n=32)** |  |  |  |
| **cfDNA (ng/mL)** | 1541.1; 1452.0-1683.3 | 1598.7; 1489.6-1679.4 | 1904.0; 1619.8-2191.7 | NS | **<0.001** | **0.014** |
| **Nucleosomes (AU)** | 0.09; 0.05-0.16 | 0.10; 0.05-0.16 | 0.10; 0.04-0.19 | NS | NS | NS |
| **citH3 (AU)** | 0.51; 0.40-0.67 | 0.81; 0.51-1.29 | 0.79; 0.44-1.13 | **0.019** | **0.049** | NS |
| **Calprotectin (ng/mL)** | 1286.1; 836.0-1835.8 | 1372.1; 755.1-2259.4 | 2694.5; 1995.8-3953.6 | NS | **<0.001** | **<0.001** |
| **MPO (ng/mL)** | 50.8; 42.7-60.4 | 50.9; 37.2-63.6 | 55.5; 40.5-83.65 | NS | NS | NS |
